# Acute Lesion Imaging in Predicting Chronic Tissue Injury in the Ventricles

**DOI:** 10.3389/fcvm.2021.791217

**Published:** 2022-01-28

**Authors:** Abdel Hadi El Hajjar, Chao Huang, Yichi Zhang, Mario Mekhael, Charbel Noujaim, Lilas Dagher, Saihariharan Nedunchezhian, Christopher Pottle, Eugene Kholmovski, Tarek Ayoub, Aneesh Dhorepatil, Michel Barakat, Takano Yamaguchi, Mihail Chelu, Nassir Marrouche

**Affiliations:** ^1^Tulane Research Innovation for Arrhythmia Discoveries, Tulane University School of Medicine, New Orleans, LA, United States; ^2^Department of Biomedical Engineering, Johns Hopkins University, Baltimore, MD, United States; ^3^Department of Cardiology, PeaceHealth, Bellingham, WA, United States; ^4^Department of Cardiology, Saga University, Saga, Japan; ^5^Baylor Heart Clinic, Baylor College of Medicine, Houston, TX, United States

**Keywords:** arrhythmia, ventricular ablation, catheter ablation, animal model, electrophysiology

## Abstract

**Background:**

Chronic lesion formation after cardiac tissue ablation is an important indicator for procedural outcome. Moreover, there is a lack of knowledge regarding the features that predict chronic lesion formation.

**Objective:**

The aim of this study is to determine whether acute lesion visualization using late gadolinium enhanced magnetic resonance imaging (LGE-MRI) can reliably predict chronic lesion size.

**Methods:**

Focal lesions were created in left and right ventricles of canine models using either radiofrequency (RF) ablation or cryofocal ablation. Multiple ablation parameters were used. The first LGE-MRI was acquired within 1–5 h post-ablation and the second LGE-MRI was obtained 47–82 days later. Corview software was used to perform lesion segmentations and size calculations.

**Results:**

Fifty-Five lesions were created in different locations in the ventricles. Chronic volume size decreased by a mean of 62.5 % (95% CI 58.83–67.97, *p* < 0.0005). Similar regression of lesion volume was observed regardless of ablation location (*p* = 0.32), ablation technique (*p* = 0.94), duration (*p* = 0.37), power (*p* = 0.55) and whether lesions were connected or not (*p* = 0.35). There was no significant difference in lesion volume reduction assessed at 47–54 days and 72–82 days after ablation (*p* = 0.31). Chronic lesion volume was equal to 0.32 of the acute lesion volumes (R^2^ = 0.75).

**Conclusion:**

Chronic tissue injury related to catheter ablation can be reliably modeled as a linear function of the acute lesion volume as assessed by LGE-MRI, regardless of the ablation parameters.

## What's New?

- Lesion volume regression following catheter ablation follow the same pattern regardless ablation method and ablation parameters.
- The acute lesion assessed by LGE-MRI can be used to predict the volume of the chronic lesion.
- Chronic Lesion volume can be predicted from the acute lesion obtained after ablation using the following formula: Chronic Lesion Volume (cm3) = 0.32 * Acute Lesion Volume (cm3).
- The lesion created with catheter ablation does not reduce in size after 7.5 weeks following ablation.

## Introduction

Radiofrequency ablation plays an important role in the treatment of cardiac arrhythmias (1, 2). Several different ablation parameters such as power, duration (3, 4), contact force (5, 6) and irrigation flow rate (7–9) have been used and finetuned to increase chronic lesion formation. These settings have been studied in both human (10) and animal (11) models. However, what ultimately determines the long-term efficacy of a delivered radiofrequency (RF) ablation or cryoablation to the tissue is the chronic lesion volume (11). As acute inflammation and edema subsides after ablation, lesion size and depth often shrink, potentially creating ablation scars that most often incontiguous. Ghafoori et al. has attempted to characterize the acute imaging predictors of a chronic lesion volume (11). Therefore, we aim to compare the prediction power of acute lesion dimensions created in animals and observed on LGE-MRI as compared to standard ablation parameters and its impact on chronic lesion formation.

## Methods

### Animal Preparation

The study was approved by the Institutional Animal Care and Use Committee and conforms to the Guide for the Care and Use of Laboratory Animals. Eight adult (25–30 kg), male, class A purpose-bred mongrel dogs were used for the study. Induction of anesthesia was performed through intravenous injection of propofol (0.1–8 mg/kg). Deep anesthesia was maintained with isoflurane (1.5–3.5%) under mechanical ventilation.

### Radiofrequency Ablation, Cryofocal Ablation and Electroanatomic Mapping

Right and left femoral vein access were obtained percutaneously. An 11 Fr introducer was placed in the left femoral vein for advancing intra-cardiac echocardiogram (ICE) catheter and a 16 Fr introducer were placed in the right femoral vein for advancing all mapping and ablation catheters. When left heart access was required, the ICE catheter (Biosense Webster, USA) was advanced to the right atrium to visualize the inter-atrial septum. Under fluoroscopy (Artis Zeego; Siemens USA, Malvern, PA) and ICE guidance, a transseptal puncture was performed and the 8.5 Fr sheath (St Jude Medical, USA) was advanced to the left atrium to achieve left heart access.

All subjects underwent high-density electroanatomical mapping (Biosense Webster, CARTO, USA) in the right and left ventricles before and after each ablation lesion. All ablation lesions were delivered utilizing radiofrequency (RF) energy (THERMOCOOL SMARTTOUCH® SF catheter, Biosense Webster, USA) or cryoablation (Freezor MAX, Medtronic, USA). Sheaths were exchanged as needed to accommodate the sizes of the ablation catheters and ablation locations. Steerable sheaths included Agilis NxT Steerable Introducer (St Jude Medical, USA), FlexCath Advance (Medtronic, USA), and Oscor Destino (Oscor Inc, USA).

Animals underwent multiple RF and cryo ablations in the right and left ventricles at both the initial and final catheter-based sessions. We used multiple ablation parameters to perform RF ablation: power ranged from 30 to 50 W, duration from 10 to 30 sec and contact forces were superior to 10 g. Duration of cryo ablation ranged from 90 to 120 seconds (Supplementary Table 1). Contiguous lesions were performed by ablating a specific point then retracting the catheter and ablating next to the first one. The distribution of the different lesions was detailed in the Supplementary Table.

### Timeline of Procedures

Subjects underwent the following study sessions: a baseline cardiac MRI scan and the initial catheter-based mapping/ablation/MRI session 1 week later. In less than an hour following the last lesion delivery cardiac MRI was started. Around seven weeks after the initial ablation, a follow-up MRI was performed before the animals were euthanized. Moreover, a post-mortem MRI scan was performed, and the heart was removed and treated. Baseline cardiac MRI was performed to assess for initial tissue characteristics. The MRI performed after lesion delivery evaluated acute lesion formation, and the follow-up MRI evaluated chronic lesion formation. A post-mortem MRI was necessary to correlate imaging findings with histology.

### MRI of Ablation Lesions

#### MRI Studies

All MRI studies were performed on 3 Tesla MRI scanners (Verio or Prisma; Siemens Healthcare, Erlangen, Germany) using body and spine phased-array coils. Baseline imaging protocol included T1 and T2 mapping, double inversion recovery (DIR) prepared T2-weighted (T2w) turbo-spin echo (TSE) sequence, 3D T1-weighted (T1w) sequence, contrast-enhanced MR angiography (Gd-BOPTA, 0.15 mmol/kg of gadoteridol, Bracco Diagnostic Inc., Princeton, NJ) and post-contrast 3D T1w and 3D late gadolinium enhancement (LGE) sequences. LGE scans were repeated at different time points after contrast injection.

#### Scan Parameters

The parameters for the different MRI scans were as follows. Non-contrast T1w - respiratory navigated, ECG triggered, saturation recovery (SR) prepared turbo-FLASH sequence with repetition time (TR) = 3.1 ms, echo time (TE) = 1.4 ms, flip angle (FA) = 12°, inversion time (TI) = 400 ms, voxel size = 1.25 x 1.25 x 2.5 mm, fat saturation. DIR T2w TSE - respiratory navigated, ECG triggered, DIR prepared 2D TSE pulse sequence with TE = 81 ms, TR = 3 cardiac cycles, echo train length = 21, fat suppression using SPAIR, in-plane resolution of 1.25 x 1.25 mm, slice thickness of 4 mm. Contrast-enhanced MRA - respiratory navigated, ECG triggered, SR prepared 3D turbo-FLASH with resolution = 1.25 x 1.25 x 2.5 mm, TR/TE = 2.9/1.3 ms, FA = 17°, TI = 120 ms, fat saturation. Post-contrast T1w - respiratory navigated, ECG triggered, SR prepared 3D turbo-FLASH with resolution = 1.25 x 1.25 x 2.5 mm, TR/TE = 3.1/1.4 ms, FA = 15°, and TI = 150 ms. LGE - respiratory navigated, ECG triggered, inversion recovery (IR) prepared 3D turbo-FLASH with resolution = 1.25 x 1.25 x 2.5 mm, TR/TE = 3.1/1.4 ms, flip angle = 14°, TI = 230 = 330 ms, fat saturation.

### Histological Evaluation of Ablation Lesions

We obtained full-thickness biopsies from sites in the ventricles where ablations were performed. The biopsies were subsequently paraffin embedded, sectioned and Masson's trichrome stained. Slides of full thickness sections through these ablated ventricular regions were imaged with a digital slide scanner (Axio Scan.Z1, Zeiss, Jena, Germany) equipped with a 40x lens at a lateral resolution of 0.89 μm.

For the purpose of our study, histological assessment is not performed for quantification. It was performed for validation. Thus, we randomly took a sub-sampling of all the 55 lesions to get an approximation of lesions' width and depth and correlate it with LGE-MRI.

### Analysis of Lesion Volumes

Segmentation of lesions from LGE-MRI scans were performed with custom software package (Corview, Marreck Inc., Salt Lake City, UT) using the fast growcut algorithm (12). In short, we defined gross boundaries of a lesion to be segmented by drawing seed and background pixels in and around it. The fast growcut algorithm propagates these input labels based on principles derived from cellular automata to classify all the pixels. Refinements of the segmentation can be inputted in the form of additional seeds and background pixels by the user until the lesion is sufficiently segmented. Using this tool, volumes of segmented lesions at the acute and chronic stage were measured. The inter-observer and intra-observer correlation of this method was previously assessed (13).

Lesion segmentation and volume calculation were performed by two independent individuals trained on the Corview software. The Bland-Altman plot of both measurements is included as a Supplementary Figure. When a significant disagreement was found between the two observers, they discussed the segmentation and reached an agreement on what optimal lesion volume measure will be used for the analysis.

### Statistical Analysis

To determine lesion size regression, we hypothesized that reduction in size was >50%. Since lesion size reduction is not normally distributed, the one-sample Wilcoxon signed-rank test was used to estimate lesion size reduction. Sub-group analysis was performed by comparing the average volume reduction in each subgroup to the lesion volume reduction across all lesions. A Mann-Whitney U test was performed (also known as the Wilcoxon rank-sum test) due to non-normal distributions of the differences.

### Predictive Model for Chronic Lesion Size

A predictive linear regression model was developed using chronic lesion size as the dependent variable. Potential independent variables were chosen using a forward selection process and internally tested for accuracy with a 5-fold cross-validation method repeated 5 times. The best model for each of the k most significant predictors (k = 1,2,3,4) were chosen based on a combination of high R^2^ and low root mean square error (RMSE).

Independent variables available for selection are the following: Chamber of the heart (categorical, Left Ventricle or Right Ventricle), Location (categorical, Free Wall or Septum), Acute lesion size (continuous), Days between measurements (continuous), Energy Source (categorical, Radial Frequency or Cryogenic), Connected lesions (categorical, yes or no) and Segmenter (categorical, two choices since the segmentation was performed by two operators). It was estimated that up to four independent variables included could explain at least 60 percent of the variation in chronic lesion size, ie. R^2^ = 0.6. The sample size of 55 was able to provide over 90% power to detect such a difference.

## Results

### Baseline Characteristics

Fifty-Five lesions were created: with 25, 14 and 16 lesions in the Left Ventricle (LV), Right Ventricle (RV) and septum, respectively. Twelve lesions were created by cryo ablation, while other lesions were obtained with RF ablation. Eight RF lesions were part of a subset of paired lesions, which formed a contiguous scar; other lesions were isolated, focal lesions. All cryo lesions were created with a duration of 90–120 seconds. The average ablation parameters used for radiofrequency lesions were power of 41.8 W, duration of 22 seconds, contact force of 13.15 g and temperature of 33.7°C. On average, follow-up MRI was performed 61.1 days after ablation.

Ablation parameters are summarized in Table 1.

**Table 1:** Characteristics of all lesions created.

**Lesion location**	**Free Wall**		**Septum**	**Average**
	**LV**	**RV**		
Count of lesions	25	14	16	55
Cryofocal	4	3	5	12
Radiofrequency	21	11	11	43
Connected lesions	4	2	2	8
Power average (W)	42.21	43.18	40	41.88
Duration average (s)	39.13	43.29	53.13	44.45
Radiofrequency	22.11	22.36	22.73	22.34
Cryofocal	120	120	120	120
Force average (g)	14.38	13.09	10.43	13.15
Temperature average©	33.25	35	32.71	33.71
Follow-up MRI (Days)	62.2	56.14	63.75	61.11

*LV, Left Ventricle; RV, Right Ventricle; MRI, Magnetic Resonance Imaging*.

### Chronic Lesion Volume Regression

**Figure 1:**
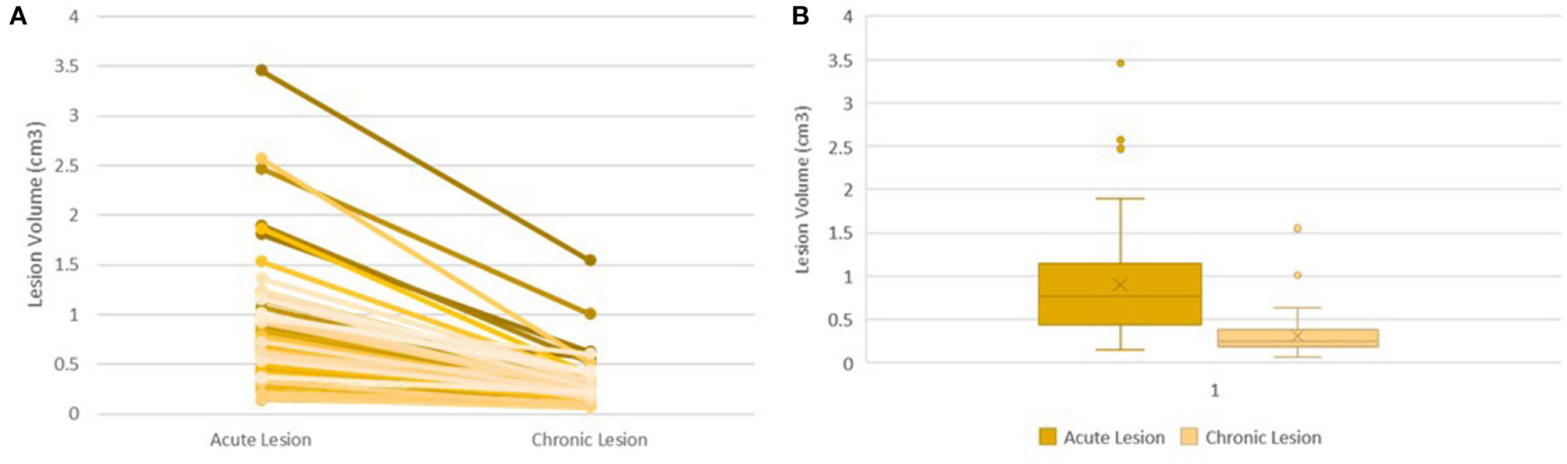
Lesion Volume Evolution Following Ablation. Trend of Lesion Volume Evolution between the Acute and Chronic Phase in all 55 lesions **(A)**. Box Plot Showing Distribution of Acute Volumes and Chronic Volumes **(B)**.

Acute lesion volumes ranged from 0.15 to 2.57 cm^3^. Chronic lesion volumes ranged from 0.07 to 1.55 cm^3^. The regression of lesion volumes between the acute and chronic phases was consistent across all lesions (Figure 1A). On average, lesion volume changed from 0.90 to 0.31 cm^3^ reducing by 62.53% from the acute to chronic phase (95% CI 58.83–67.97%, *p* < 0.0005) (Figure 1B).

**Figure 2:**
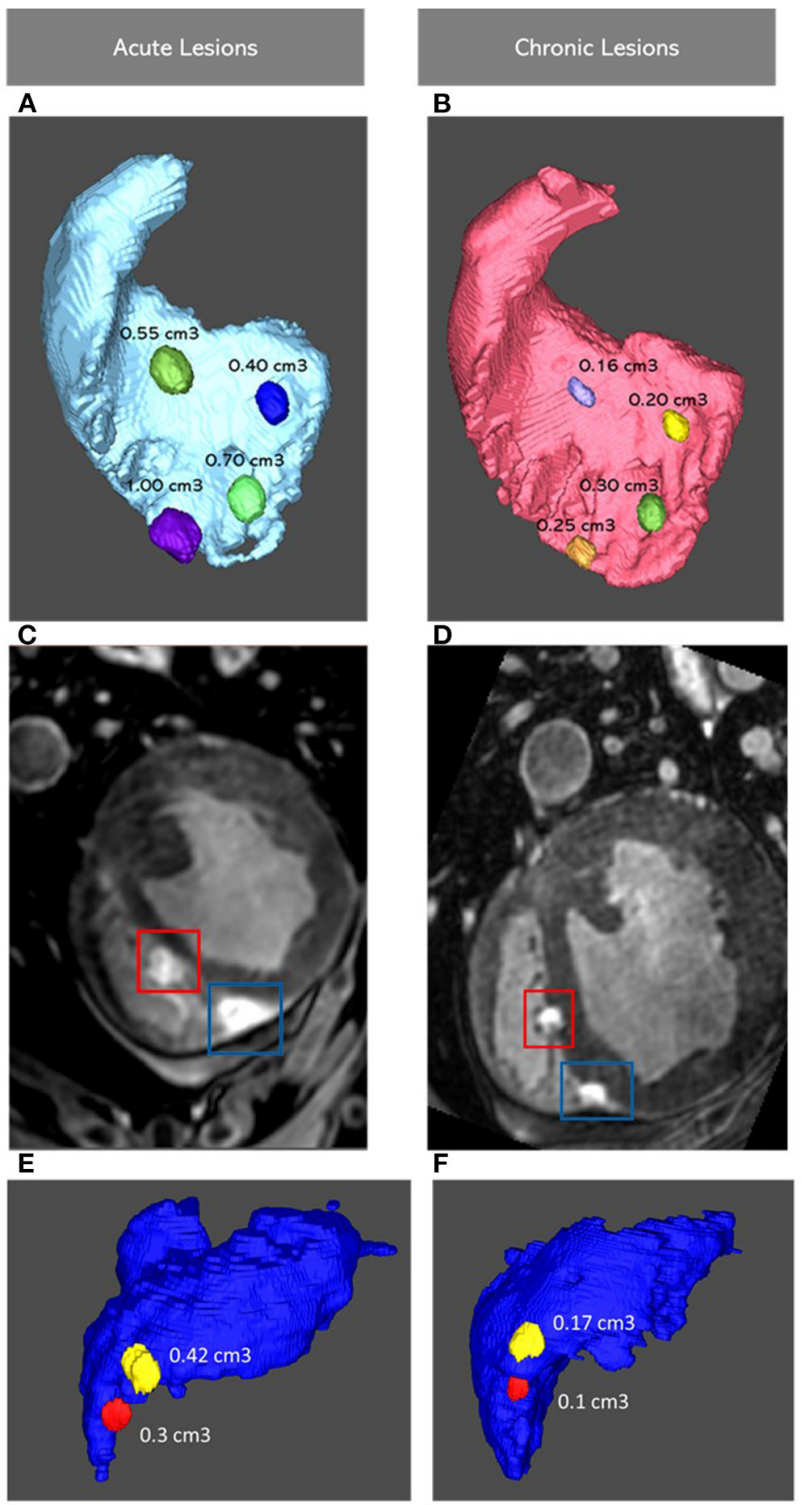
Images Showing four Lesions Represented on Corview in the RV Septum in the Acute **(A)** and Chronic **(B)** Settings, with corresponding LGE-MRI Images of the Lower Two Lesions **(C,D)**. Images Showing two Lesions in The RV Free Wall **(E,F)**. RV: Right Ventricle; LGE-MRI: Late Gadolinium Enhancement Magnetic Resonance Imaging.

Representation of lesions, acute and chronic, on Corview can be seen in Figure 2. Representative LGE-MRI images of acute and chronic lesions are shown in Figures 2C,D.

### Histological Assessment of Lesions

Twenty-three lesions were assessed pathologically for width, depth and transmurality. Lesion dimensions seen on LGE-MRI were very similar to lesion dimensions assessed on histology. Four lesions showed transmurality in both LGE-MRI and pathological assessment. Two transmural lesions were created in the RV free wall using 35W-30S and the two others were created in the LV apex using 50W-20S.

On histology, average lesion width was 6.1 mm and average lesion depth was 4.95 mm. On LGE-MRI, average lesion width was 6.2 mm and average lesion width was 5.02 mm. There was no significant difference in both lesion width (*p* = 0.97) and lesion depth (*p* = 0.74) between histological assessment and LGE-MRI.

**Figure 3:**
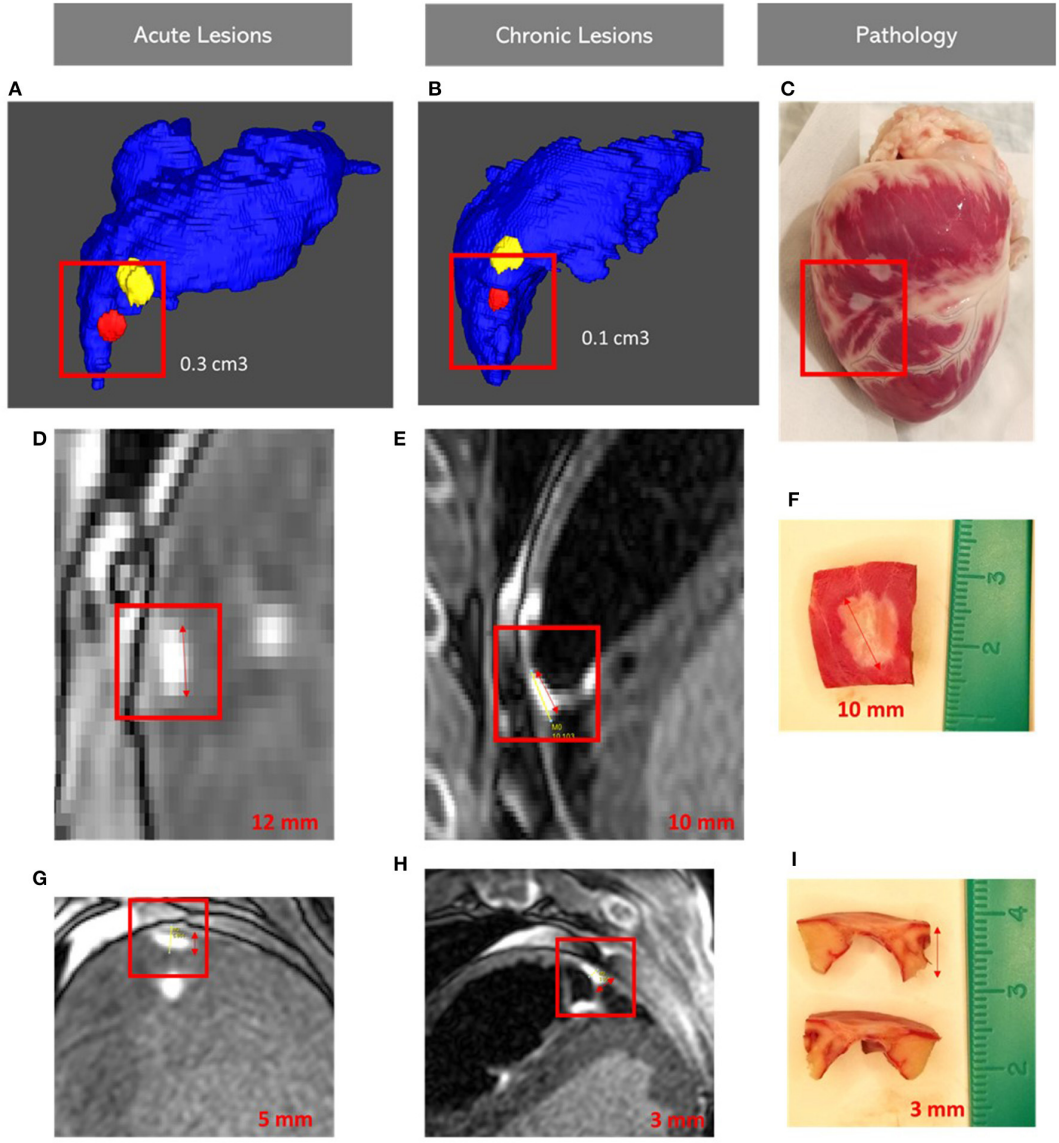
Images showing lesion volumes in both acute and chronic phases in the right ventricular free wall: Acute **(A)**, Chronic corview representation **(B)** and on gross pathology **(C)**; Acute **(D,G)**, Chronic LGE-MRI **(E,H)** and histology **(F,I)**. LGE-MRI: Late Gadolinium Enhancement Magnetic Resonance Imaging.

Measurements of the chronic lesions obtained by LGE-MRI were consistent with histological measurements Figure 3.

Comparison of lesion measurements obtained using LGE-MRI and histological assessment is shown in Supplemental Table 2.

### Chronic Lesion Volume Regression Sub-group Analysis

**Figure 4:**
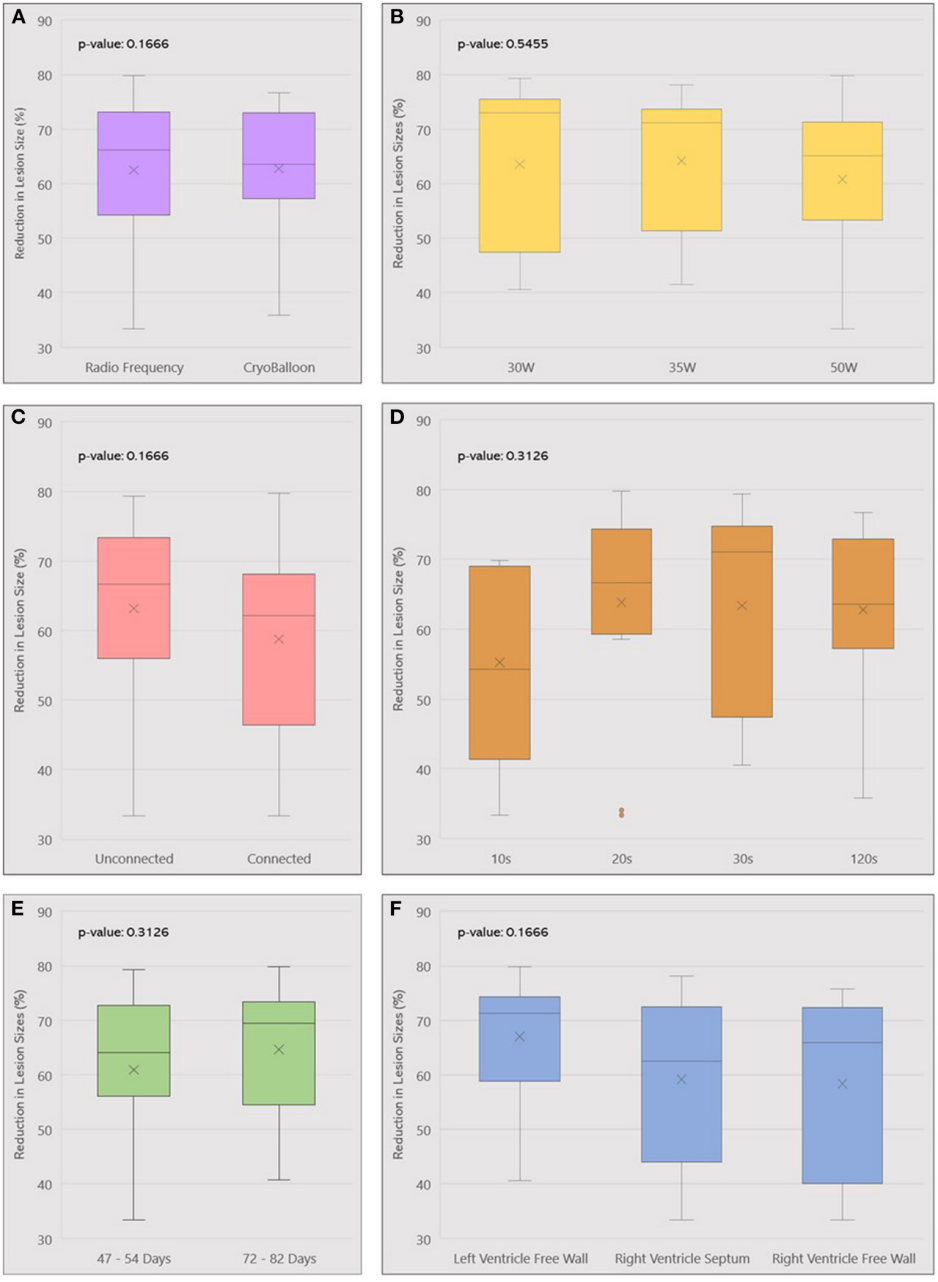
Box plot summarizing lesion volume reduction across different ablation parameters used: ablation method **(A)**, Power **(B)**, Lesion contiguity **(C)**, Duration **(D)**, Follow-up **(E)**, and Location **(F)**. W, Watts; s, seconds.

There was no difference in lesion regression trends between ablation methods. Cryofocal lesions reduced by 62.76% ± 12.79 at the chronic phase and RF lesions reduced by 62.47% ± 13.92 (*p* = 0.94). A consistent trend of lesion regression was observed across all locations, including LV Free Wall (reduction in lesion size by 67.05 ± 10.04%), RV Free Wall (58.34 ± 16.68%), and Septum (59.15 ± 14.06%) (*p* = 0.17). The same trend was observed regardless of different ablation power (reduction in lesion size with 30, 35, and 50W was respectively 63.51 ± 14.88%, 64.22 ± 13.52% and 60.78 ± 14.26%, *p* = 0.55) or ablation duration (*p* = 0.38). Subgroup analysis is summarized in Figure 4.

### Timeline of Chronic LGE-MRI

**Figure 5:**
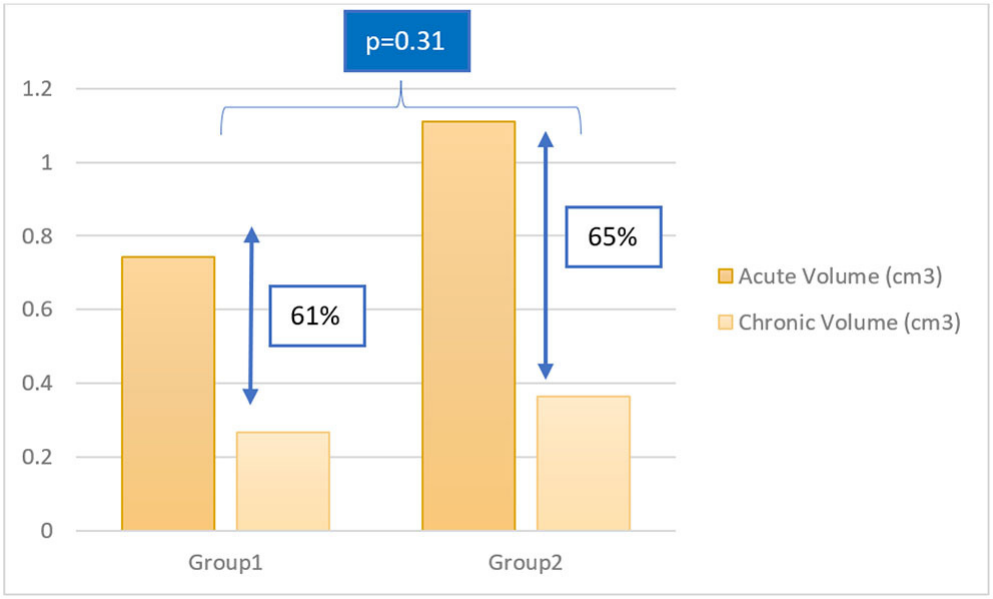
Column chart of acute and chronic lesion volumes in group 1 and group 2. Group 1: follow up in 47–54 days; Group 2: follow up in 72–82 days.

Chronic MRI was obtained at different timepoints post-ablation. Follow up durations between lesion creation and chronic MRI varied from 47 to 82 days. We divided chronic lesions in two groups based on time interval between ablation and chronic MRI: group 1 had a follow up in 47–54 days (*n* = 31) while group 2 had a follow-up in 72–82 days (*n* = 24). Average volumes of acute and chronic lesions for both groups are shown in Figure 5. There was no significant difference in lesion size reduction between the two groups: in group 1, lesions decreased by 60.91 ± 14.50%. In group 2, lesions decreased by 64.63 ± 12.25% (*p* = 0.31).

### Predictive Model

The best model for each of the k most significant predictors (k = 1,2,3,4) were chosen based on a combination of high R^2^ and low root mean square error (RMSE). The results are summarized in Table 2. The adjusted R^2^ and MSE figures are given for the multiple regression model using the entire set.

**Table 2:** Adjusted R^2^ and model MSE for each predictor's subset.

**Predictor**	**Predictor variables**	**Adjusted R^**2**^**	**Model MSE**
1	Acute Size (AS)	0.75	0.014
2	AS + Lesions Connected (LC)	0.77	0.012
3	AS + LC + Heart Chamber (HC)	0.77	0.012
4	AS + LC + HC + Duration	0.77	0.012

*MSE, Mean Square Error*.

Acute lesion volume calculated on LGE-MRI is the best predictor of chronic lesion volume (R^2^ = 07463). Given that all lesions were created with a temperature range between 26 and 41°C, and a contact force range from 8 to 29 g, the addition of any other ablation parameters to the model did not increase its predictive value. Our model showed that chronic lesion volume estimation can be obtained using the following formula:

*Chronic Lesion Volume (cm*^3^*)* = *0.3228* * *Acute Lesion Volume (cm*^3^*)*

## Discussion

Our study showed that lesion volume regression follows the same trend regardless of ablation parameters or energy source applied during the ablation procedure. Chronic lesion volume regresses to approximately one-third of its original size as assessed by LGE-MRI and confirmed by necropsy. Moreover, no change in lesion size was detected on LGE-MRI 7.5 weeks post ablation. This is an important novel finding to help timing of follow to assess for durable ablation lesions.

### Why Does Lesion Volume Decrease With Time?

During radiofrequency ablation, high temperatures injure tissue and blood vessels, resulting in an area of microvascular obstruction (14). In the surrounding tissue, edema forms and can temporarily disrupt electrical conduction (15). Edema tends to resorb with time, thus explaining the reduction of the acute lesion volume. Ghafoori et al. showed that LGE-MRI obtained 26 min after contrast administration can predict chronic lesion volume, with the dark center of the lesion on LGE-MRI corresponding to microvascular obstruction and correlating with the final scar after edema resorption (11). However, this effect was not analyzed for cryofocal ablation. In addition, the area of microvascular obstruction tends to decrease with time, as observed in our study. Edema resorption is relevant clinically, as failure to create a contiguous, permanent scar after catheter ablation is associated with arrhythmia recurrence (16).

### Lesion Volume Regression: The Same Trend Regardless of Energy Source or Ablation Parameters

The lesion formation mechanism in cryofocal ablation is different from that of RF ablation. Here, direct cell injury occurs from cooling of extracellular space, leading to a hyper osmotic environment. This leads to a fluid shift from the intracellular to extracellular space, resulting in reversible damage. When temperatures reach below −40°C, intracellular cooling becomes irreversible, leading to cellular death. In parallel, tissue cooling promotes vasoconstriction of blood vessels and circulation ceases uniformly, leading to ischemic necrosis. Rewarming of the cooled tissue results in a hyperemic response and increased vascular permeability, leading to edema. Accompanying endothelial damage leads to micro-thrombus of the vessels and eventual irreversible damage. Despite differences in the mechanism of lesion creation between RF and cryo ablation, there remains a common endpoint: microvascular obstruction and edema. Therefore, the eventual evolution of the chronic lesion is expected to be similar in both techniques, as demonstrated s in our study.

Previous studies demonstrated that high contact force, higher power or higher ablation index lead to larger lesions immediately after ablation (17). Further studies with T2w MRI and histopathology showed that higher contact force results in greater degrees of edema (18). Thus, initial ablation parameters contribute to acute lesion size. It is also worthy to note that although larger lesions are associated with greater degrees of edema, the eventual resorption of edema is always proportional to the acute lesion size.

### Resistive vs. Conductive Heating: What Does Contribute to Lesion Durability?

Another important aspect in understanding lesion formation is the concept of resistive and conductive heating. Resistive heating results from the direct tissue contact with the catheter. Current delivery at the interface between the catheter tip and the tissue surface causes direct resistive heating. Conductive heating results from passive propagation of the heat wave into deeper layers of the tissue. Resistive heating is dependent on energy delivered while conductive heating is time dependent (19, 20). On the other hand, ablation duration is the determinant factor for conductive heating (20). Both resistive and conductive heating can produce irreversible tissue injury, explaining the roles of the various ablation parameters in acute lesion formation (21).

However, this concept of resistive or conductive heating does not apply the same way to edema formation or resorption. Since edema forms on the periphery of the heated tissue, its size is largely unaffected by ablation parameters (21). Consequently, the process of lesion volume regression, which is characterized mainly by edema resorption, is also independent of ablation parameters, as supported by our study findings.

### Real-Time Imaging for Catheter Ablation

Multiple studies are examining the feasibility of imaging assisted ablation and its role in visualizing acute lesion formation (22). Real time MRI can be used to target fibrotic lesions (arrhythmogenic substrates) or visualize scar formation (23). Other imaging modalities, such as intracardiac echography (ICE) are more integrated in clinical practice (24).

However, real time imaging can only help assess acute lesion volume. The subsequent lesion shrinkage may result in arrhythmia recurrence and catheter ablation failure (25). One future avenue is to observe if this model can be applied in human hearts following ablation. Therefore, the model can be integrated with real time imaging guided catheter ablation to help reliably account for lesion volume regression. Furthermore, physicians would be able to visualize a simulated representation of the chronic scar while performing ablation.

In addition, the ideal timeline for a follow-up imaging to visualize the scar is not established. Many clinicians tend to schedule a follow-up cardiac MRI around 3 months after ablation. Our findings suggest that follow-up MRI can be performed as early as 6 weeks following ablation.

## Limitations

This present study has several limitations. First, all lesions were studied using LGE-MRI. LGE-MRI is not optimal in visualizing edema and is inferior to other imaging modalities such as T1w MRI in assessment of acute RF lesions (11, 12). However, in the chronic settings, lesions are composed of only scar tissue and LGE-MRI successfully assess chronic lesion size. Second, some lesions were not well demarcated, potentially leading to biases in the segmentation process. To tackle this issue, two independent operators segmented the lesions, and the results were reconciled. Third, ablation was performed point by point, and thus, our results may not be generalizable to all ablation techniques (such as dragging). Fourth, even if correlation between imaging and histology has been described in the literature (26), more studies are needed to increase quantification accuracy of imaging. Finally, the average temperature used for ablation in this study is lower than the usual temperature used in human catheter ablation. Future studies should test the accuracy of our predictive model in human experiments, where catheter ablation is performed using higher temperatures.

## Summary

Accurate estimation of the chronic lesion size is instrumental for ensuring the contiguity of catheter ablation scarring. This current study presents a model for chronic lesion size based only on acute lesion size as assessed by LGE-MRI immediately following ablation. Chronic lesion volume consistently reduced to approximately one-third of the acute lesion volume. Various ablation parameters, when applied within a reasonable clinical range, ultimately does not affect chronic lesion volume.

## Data Availability Statement

The raw data supporting the conclusions of this article will be made available by the authors, without undue reservation.

## Ethics Statement

The animal study was reviewed and approved by Institutional Animal Care and Use Committee at the University of Utah.

## Author Contributions

AE and CH came up with the idea, performed the analysis, and wrote the manuscript. YZ, MM, CN, LD, SN, TA, and AD helped with the analysis, participated in discussions, and proofread the manuscript. CP performed the statistical analysis. MB, TY, MC, and EK helped with procedures performed on animals. NM supervised the entire project. All authors contributed to the article and approved the submitted version.

## Funding

This work was partly supported by Biosense Webster and partly by Medtronic research grants.

## Conflict of Interest

NM reports receiving grant support and consulting fees from Abbott, Medtronic, Biosense Webster, Boston Scientific, and Siemens, receiving consulting fees from Preventice, and holding equity Cardiac Design. The remaining authors declare that the research was conducted in the absence of any commercial or financial relationships that could be construed as a potential conflict of interest.

## Publisher's Note

All claims expressed in this article are solely those of the authors and do not necessarily represent those of their affiliated organizations, or those of the publisher, the editors and the reviewers. Any product that may be evaluated in this article, or claim that may be made by its manufacturer, is not guaranteed or endorsed by the publisher.

## Abbreviations

LGE-MRI: Late Gadolinium Enhancement Magnetic Resonance Imaging
RF: Radiofrequency
Cryo: Cryofocal
EP: Electrophysiology
ICE: Intra-cardiac Echocardiogram
EAM: Electroanatomic Mapping
LV: Left Ventricle
RV: Right Ventricle.
