# Supplementary material for: Acute Lesion Imaging in Predicting Chronic Tissue Injury in the Ventricles
Source: Front Cardiovasc Med. 2022 Jan 28;8:791217. doi: 10.3389/fcvm.2021.791217 (PMC8831749; doi:10.3389/fcvm.2021.791217)
Supplement: Supplementary file 1 [file Table_1.docx]

**Supplementary table 1:** Distribution of the 55 lesions and corresponding ablation parameters. *N/A: not applicable. X: missing data. RV: Right ventricle. LV: Left ventricle.*

| **Canine ID** | **Lesion Chamber** | **Location** | **Energy source** | **Duration** | **Power** | **Temperature** | **Force** |
| --- | --- | --- | --- | --- | --- | --- | --- |
| **Canine #1** | RV | free wall | RF | 30 | 35 | 35 | 15 |
|  | RV | septum | RF | 30 | 35 | 31 | 11 |
|  | LV | free wall | RF | 28 | 43 | 34 | 15 |
| **Canine #2** | LV | free wall | RF | 18 | 43 | 35 | 16 |
|  | RV | septum | RF | 30 | 35 | 34 | 19 |
|  | RV | free wall | RF | 30 | 35 | 35 | 16 |
|  | RV | free wall | RF | 30 | 35 | 40 | 9 |
|  | LV | free wall | RF | 30 | 35 | 35 | 18 |
|  | RV | free wall | RF | 30 | 35 | 31 | 29 |
|  | RV | free wall | RF | 30 | 35 | 34 | 22 |
| **Canine #3** | RV | free wall | RF | 8 | 50 | 41 | 8 |
|  | LV | free wall | RF | 10 | 50 | 34 | 18 |
|  | RV | septum | RF | 10 | 50 | 39 | 4 |
|  | LV | free wall | RF | 10 | 50 | 34 | 18 |
|  | RV | free wall | RF | 8 | 50 | 38 | 10 |
|  | RV | septum | RF | 10 | 50 | 39 | 4 |
|  | LV | free wall | RF | 10 | 50 | 39 | 10 |
|  | LV | free wall | RF | 10 | 50 | 32 | 21 |
| **Canine #4** | RV | free wall | RF | 20 | 50 | 34 | 10 |
|  | RV | septum | RF | 10 | 50 | 33 | 15 |
|  | RV | free wall | RF | 20 | 50 | 31 | 11 |
|  | RV | free wall | RF | 20 | 50 | 34 | 8 |
|  | RV | free wall | RF | 20 | 50 | 32 | 6 |
|  | LV | free wall | RF | 20 | 50 | 36 | 11 |
|  | LV | free wall | RF | 20 | 50 | 35 | 12 |
|  | LV | free wall | RF | 20 | 50 | 33 | 12 |
|  | LV | free wall | RF | 20 | 50 | 33 | 8 |
| **Canine #5** | LV | free wall | RF | 20 | 57 | 27 | 14 |
|  | LV | free wall | RF | 20 | 50 | 26 | 15 |
|  | RV | septum | RF | 20 | 50 | 27 | 8 |
|  | RV | septum | RF | 20 | 50 | 26 | 12 |
|  | LV | free wall | RF | 20 | 50 | 27 | 13 |
| **Canine #6** | RV | septum | Cryo | 120 | N/A | N/A | N/A |
|  | RV | septum | Cryo | 120 | N/A | N/A | N/A |
|  | RV | septum | Cryo | 120 | N/A | N/A | N/A |
|  | RV | septum | Cryo | 120 | N/A | N/A | N/A |
|  | RV | free wall | Cryo | 120 | N/A | N/A | N/A |
|  | LV | free wall | RF | 30 | 30 | 36 | 19 |
|  | LV | free wall | RF | 30 | 30 | 35 | 14 |
|  | LV | free wall | RF | 30 | 30 | 35 | 14 |
|  | LV | free wall | RF | 30 | 30 | 35 | 13 |
| **Canine #7** | RV | septum | RF | 30 | 30 | X | X |
|  | RV | septum | RF | 30 | 30 | X | X |
|  | RV | septum | RF | 30 | 30 | X | X |
|  | RV | septum | RF | 30 | 30 | X | X |
|  | LV | free wall | Cryo | 120 | N/A | N/A | N/A |
|  | LV | free wall | Cryo | 120 | N/A | N/A | N/A |
|  | LV | free wall | Cryo | 120 | N/A | N/A | N/A |
|  | LV | free wall | Cryo | 120 | N/A | N/A | N/A |
| **Canine #8** | RV | septum | Cryo | 120 | N/A | N/A | N/A |
|  | RV | free wall | Cryo | 120 | N/A | N/A | N/A |
|  | RV | free wall | Cryo | 120 | N/A | N/A | N/A |
|  | LV | free wall | RF | 30 | 30 | X | X |
|  | LV | free wall | RF | 30 | 30 | X | X |
|  | LV | free wall | RF | 30 | 30 | X | X |
