# Supplementary material for: Acute Lesion Imaging in Predicting Chronic Tissue Injury in the Ventricles
Source: Front Cardiovasc Med. 2022 Jan 28;8:791217. doi: 10.3389/fcvm.2021.791217 (PMC8831749; doi:10.3389/fcvm.2021.791217)
Supplement: Supplementary file 2 [file Table_2.docx]

**Supplementary table 2:** Comparison of lesion measurements obtained using LGE-MRI and histological assessment. Lesion width and depth are in mm; LGE-MRI: late gadolinium enhancement magnetic resonance imaging.

|  | Histology | LGE MRI | P value |
| --- | --- | --- | --- |
| Lesion Width | | | |
| Mean | 6.213 | 6.22 | - |
| Standard Deviation | 2.414 | 1.664 | - |
| Standard Error Mean | 0.670 | 0.462 | - |
| Paired Difference Mean | -0.010 [95% CI: (-0.607, 0.588)] | | 0.973 |
| Paired Difference Standard Deviation | 0.989 | | |
| Paired Difference Standard Error Mean | 0.274 | | |
| Lesion Depth | | | |
| Mean | 4.95 | 5.02 | - |
| Standard Deviation | 2.174 | 1.585 | - |
| Standard Error Mean | 0.628 | 0.458 | - |
| Paired Difference Mean | -0.075 [95% CI : (-0.564, 0.414)] | | 0.741 |
| Paired Difference Standard Deviation | 0.769 | | |
| Paired Difference Standard Error Mean | 0.222 | | |
