# Supplementary material for: Acute Lesion Imaging in Predicting Chronic Tissue Injury in the Ventricles
Source: Front Cardiovasc Med. 2022 Jan 28;8:791217. doi: 10.3389/fcvm.2021.791217 (PMC8831749; doi:10.3389/fcvm.2021.791217)

**Supplementary Figure:** Bland-Altman plot showing inter-observer variability of lesion volume measures by the two observers.


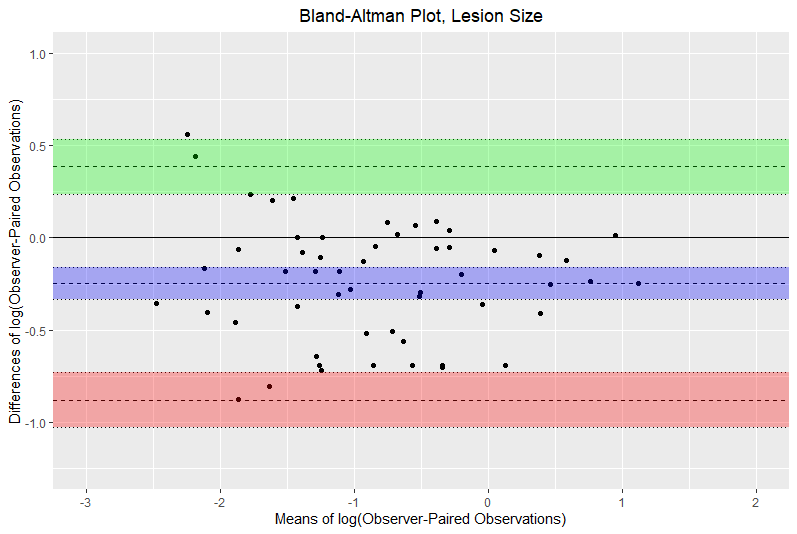

Supplement: Supplementary file 3 [file Data_Sheet_1.docx]
